# Immune Cells Profiles in the Different Sites of COVID-19-Affected Lung Lobes in a Single Patient

**DOI:** 10.3389/fmed.2022.841170

**Published:** 2022-02-16

**Authors:** Sadahiro Iwabuchi, Kyohei Miyamoto, Mayuko Hatai, Yurina Mikasa, Masahiro Katsuda, Shin-ichi Murata, Toshikazu Kondo, Hiroki Yamaue, Shinichi Hashimoto

**Affiliations:** ^1^Department of Molecular Pathophysiology, Institute of Advanced Medicine, Wakayama Medical University, Wakayama, Japan; ^2^Department of Emergency and Critical Care Medicine, Wakayama Medical University, Wakayama, Japan; ^3^Departments of Human Pathology, Wakayama Medical University, Wakayama, Japan; ^4^Second Department of Surgery, Wakayama Medical University, Wakayama, Japan; ^5^Department of Forensic Medicine, Wakayama Medical University, Wakayama, Japan

**Keywords:** COVID-19, SARS-CoV-2, immune cells, basal cells, AQP3

## Abstract

Whereas the COVID-19 disease pathophysiology is under investigation, it is important to identify the pathways of viral transmission and inflammation from the pre-illness to the disease-onset stages. We analyzed five lung lobes from a patient with COVID-19 who finally died after prolonged lung protective ventilation. Pathological examination revealed moderate inflammation in upper lung lobes and uneven yet severe inflammation and diffuse alveolar damage in lower lung lobes. SARS-CoV-2 was detected at higher levels not in severely, but rather moderately inflamed middle lung lobes, and immunohistochemistry and bulk RNA-sequencing results showed that immune cells were detected at higher levels in lower lung lobes. The mRNA expression of cytokine families varied. We found an increase in keratin 5- or aquaporin 3-expressing basal cells in the severely inflamed lower lung lobes, and the alveolar stromal tissues were filled with them. Thus, this analysis of lung samples from a patient helps to determine the COVID-19 pathophysiology at a specific time point, and the virus localization and inflammatory responses at each site of the lungs provide various important indications.

## Introduction

The coronavirus disease (COVID-19), caused by severe acute respiratory syndrome coronavirus 2 (SARS-CoV-2), is a global health crisis, but there is still limited understanding of the disease pathophysiology. Various organ tissues obtained from biopsy samples or peripheral blood samples from patients with COVID-19 have been analyzed using the innovative single-cell RNA sequencing method (1–4). These studies have investigated the systematic immune defense mechanism against SARS-CoV-2 at the single-cell level, and a comprehensive analysis has helped to obtain valuable information. Currently, there are a limited number of reports on the detection of SARS-CoV-2 and its interaction with the microenvironment in some organs studied using fresh biopsied tissue and formalin-fixed, paraffin-embedded (FFPE) specimens (5–9). In most cases, the cellular distribution of viral components, histopathological evaluation of patients with COVID-19 exhibiting different symptoms, or identification of effective SARS-CoV-2 antibodies and probes has been reported.

Here, we performed an exhaustive pathophysiological analysis of samples from a patient with COVID-19 who died after prolonged lung-protective ventilation. This comprehensive analysis indicated the distribution of SARS-CoV-2 and the cellular and molecular differences among mild-to-severely inflamed microenvironments in different lung lobes from a patient. We further found that the damaged lung lobes were filled with keratin 5- and/or aquaporin 3-expressing basal cells.

## Materials and Methods

### Samples

A 79-year-old man was admitted to our intensive care unit with respiratory failure due to COVID-19. After a 16-day course of invasive mechanical ventilation, he died due to multiple organ failure (10). Three days before death, we obtained a computed tomography scan of his lungs, which revealed worsening consolidation in the lower lungs and ground glass opacification in the upper lungs. Autopsy was performed on the day of death; in February 2020, after 5 weeks of formalin-fixation (in 10% neutral-buffered formalin) to prevent secondary infections. Upon gross examination, the weights of the right and left lungs were found to be 1,045 g and 940 g, respectively. We dissected the lung tissues from the five lobes, and representative area from each lung lobe which clearly showed the symptoms were subdivided. The fixed lung lobes were embedded in paraffin according to a standard method (FFPE).

### Immunohistochemistry (IHC)

Tissue sections (4-μm thick) were cut, dewaxed, rehydrated using xylene and graded alcohol, and stained with H&E. For Masson's trichrome staining, the sections were stained with hematoxylin, Biebrich starlet–acid fuchsin solution, and phosphomolybdic–phosphotungstic acid solution for 10–15 min, and then with aniline blue solution and 1% acetic acid solution for 5 min. The collagen fibers were stained blue, and the nuclei and background were stained black and red, respectively. IHC for each protein was performed as follows; 4-μm-thick sections were inactivated by treating with an antigen activator (pH 9.0) (DAKO: K8004) for 20 min at 95°C and probed with primary antibodies such as anti-KRT5 (Abcam, ab259429, 1:100) or anti-AQP3 antibodies (Abcam, ab125219, 1:300) overnight at 4°C. After probing with the primary antibodies, the sections were treated with mouse- or rabbit-specific anti-IgG antibodies for 30 min at 20°C and were visualized after treatment with 3,3-diaminobenzidine for 10 min at 20°C. Subsequently, the sections were counterstained with hematoxylin. All stained sections were examined under a fluorescence microscope (Keyence, BZ-X710).

For double immunofluorescent staining, a 4-μm-thick section was inactivated by treating with an antigen activator (pH 9.0) for 20 min at 95°C and treated with primary antibodies against KRT5 (1:200) overnight at 4°C. Histofine simple stain Max-PO (M) (Nichirei) was applied to the sections for 30 min at 20°C, and the sections were then stained using the TAS Fluorescein System (FITC) (AKOYA Bioscience) for 10 min at 20°C. Next, the sections were inactivated by treating with an antigen activator for 10 min at 95°C. This was followed by treatment with an anti-AQP3 antibody (1:900) for 30 min at 20°C. Histofine simple stain Max-PO (R) (Nichirei) was applied to the sections for 30 min at 20°C, following which the sections were stained with the TAS Fluorescein System (Cyanin3) (AKOYA Bioscience) for 10 min at 20°C. After DAPI staining, the sections were visualized under a BZ-710X.

### In Site Hybridization (ISH)

ISH was performed using the RNAScope 2.5 HD Duplex Reagent Kit (Advanced Cell Diagnostics), with 20 pairs of double Z proprietary RNA probes targeting the spike gene of SARS-CoV-2 (V-nCov2019-S, C1) and antisense of V-nCoV2019-S (C2). We used known RNA probes for SARS-CoV-2 spike RNA or SARS-CoV-2 negative-sense (replicative intermediate) RNA from the spike region. Briefly, FFPE sections were heated for 1 h at 60°C and deparaffinized by treating with xylene and ethanol. After air drying, the sections were treated with hydrogen peroxide at 20°C for 10 min, followed by heat-mediated retrieval using Target Retrieval Solution for 30 min at 40°C. The two probes were mixed at a ratio of 1:50 (C2:C1), and the section was treated for 2 h at 40°C, followed by four rounds of amplification with Hybridize Amp for 15–30 min at 40°C and two rounds for 15–30 min at 20°C. The C2 signals were visualized by treatment with the RED solution. The sections were subjected to two rounds of hybridization amplification for 15–30 min at 40°C, followed by two more rounds for 15–30 min at 20°C. C1 signal detection was performed using the GREEN solution for 20 min at 20°C. Finally, the sections were placed in 50% hematoxylin for 30 s at 20°C, then in 0.02% ammonia solution for 10 s, and dried for 15 min at 60°C.

### RNA Extraction From FFPE Samples

RNA was obtained from FFPE samples using the commercially available kit NucleoSpin total RNA FFPE (Marcherey-Nagel GmbH&Co.KG). In brief, two slices of thickness 10 μm were treated with Paraffin dissolver for 5 min at 56°C and with proteinase K for 90 min. After DNase treatment, approximately 50–400 ng/μL of high-purity RNA was eluted and stored at −80°C before use.

### Bulk RNA-Seq

RNA quality was evaluated using an Agilent 4200 TapeStation (Agilent Technologies), and the concentration was measured using a Qubit Fluorometer (Thermo). A total of 1,000–3,500 ng RNA from each site of lung lobes were used, and the libraries for sequencing were constructed using TruSeq Stranded mRNA (Illumina) according to the protocol. The quantity of the libraries were estimated using a KAPA Library Quantification kit (Roche). The average size of the libraries was 292–340 bp. High-throughput sequencing of the samples was performed using the NextSeq 500/550 High Output Kit v2.5 (Illumina, 75 cycles pair-end, 40/40 cycles). The average sequence reads per sample was 23,979,221. The bulk-RNA-Seq results were analyzed using the CLC Genomics Workbench Version 12.0.2 (Filgen Inc.). Target gene sets, such as the genes with highest expression in lower lobes, were analyzed using the Gene Ontology enrichment analysis tools Metascape and Coronascape (11). For SARS-CoV-2 detection, annotated RNA-Seq data were applied to GenBank data (MW400961) on a CLC Genomics Workbench. In addition, the heat map for bulk RNA-seq (1-pearson correlation, single linkage clusters) and k-mean test (Euclidean distance, # of partitions to cluster features into 15) were performed following a CLC Genomics Workbench. All datasets were deposited under the DDBJ DRA accession number DRA012049 and BioProject accession number PRJDB11612 (https://www.ddbj.nig.ac.jp/dra/index-e.html).

### Statistical Analysis

One way analysis of variance (ANOVA) was conducted for each condition, followed by an unpaired Student's *t*-test (two-tailed). Statistical analysis data were expressed in terms of mean ± standard deviation (S.D.). Statistical significance was set at *p* < 0.05.

## Results

### Pathological Characteristics of the Lungs and Detection of SARS-CoV-2

**Figure 1:**
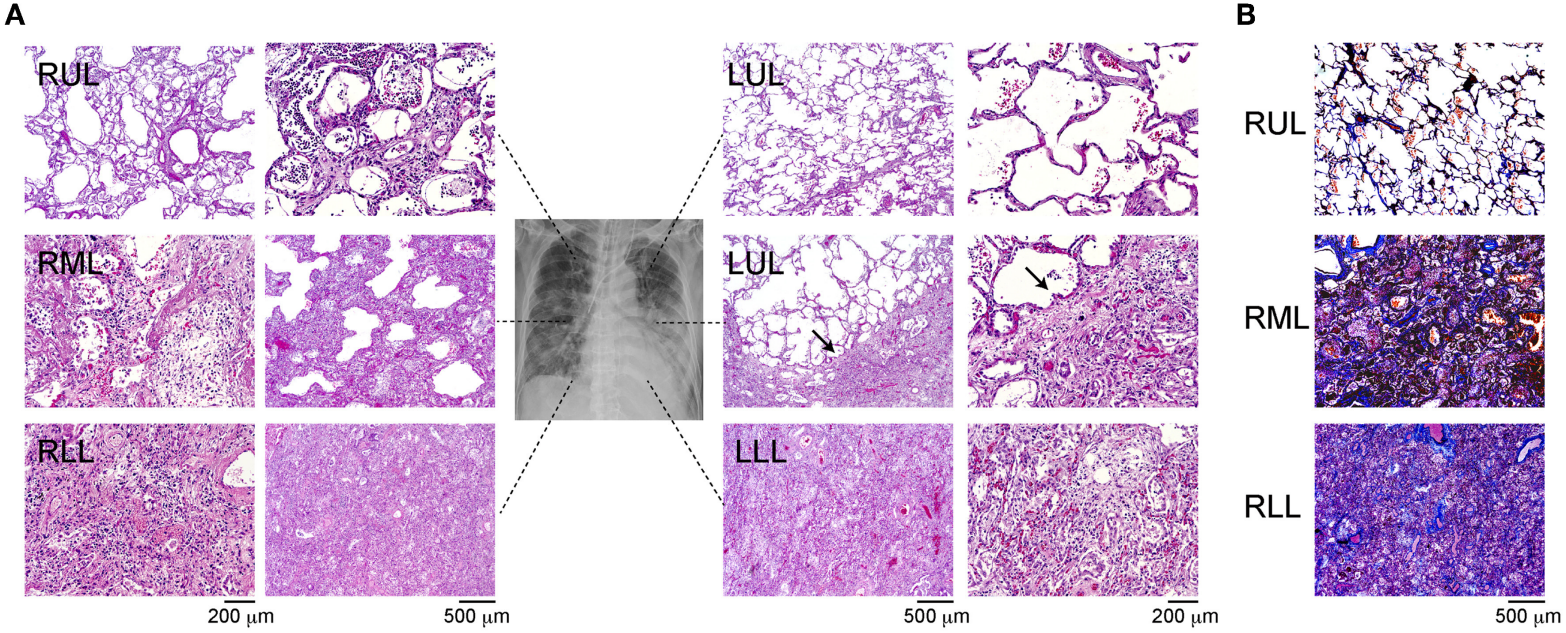
Immunohistochemistry of the different site of lung lobes. **(A)** Hematoxylin and eosin staining of lung lobes; right upper lobe (RUL), right middle lobe (RML), right lower lobe (RLL), upper and lower site of left upper lobe (LUL), left lower lobe (LLL). Mild diffuse alveolar damage (DAD) with the presence of hyaline membrane (arrow). Magnification: 200 or 500 μm. **(B)** IHC of Masson's trichrome staining from each lung lobe. The magnification bars indicate 500 μm.

The H&E-stained slides showed that barring the upper side of the left upper lobe (LUL), the other lobes showed diffuse alveolar damage (DAD) with the presence of a hyaline membrane (Figure 1A). In the right upper lobe (RUL) and lower part of the LUL, we observed mildly thickened alveolar walls and hyaline membrane formation, with the presence of swollen pneumocytes and squamous metaplasia. The alveolar spaces were filled with neutrophils in some lesions (Figure 1A, arrow), and ~25–30% of the lower regions showed mild inflammation and DAD. The characteristics of the upper lobes were indicative of the acute phase of DAD. In the lower regions, the observation of fibrous and thickened alveolar walls with intra-alveolar fibrosis, referred to as Masson bodies, was more frequent (Figure 1B). The findings of the lower lobes indicated the organizing phase of DAD. Bacterial bronchopneumonia and alveolar vascular injury, such as microthrombi, were not detected in any of the lobes. The pathological evaluation was summarized in Supplementary Table 1.

**Figure 2:**
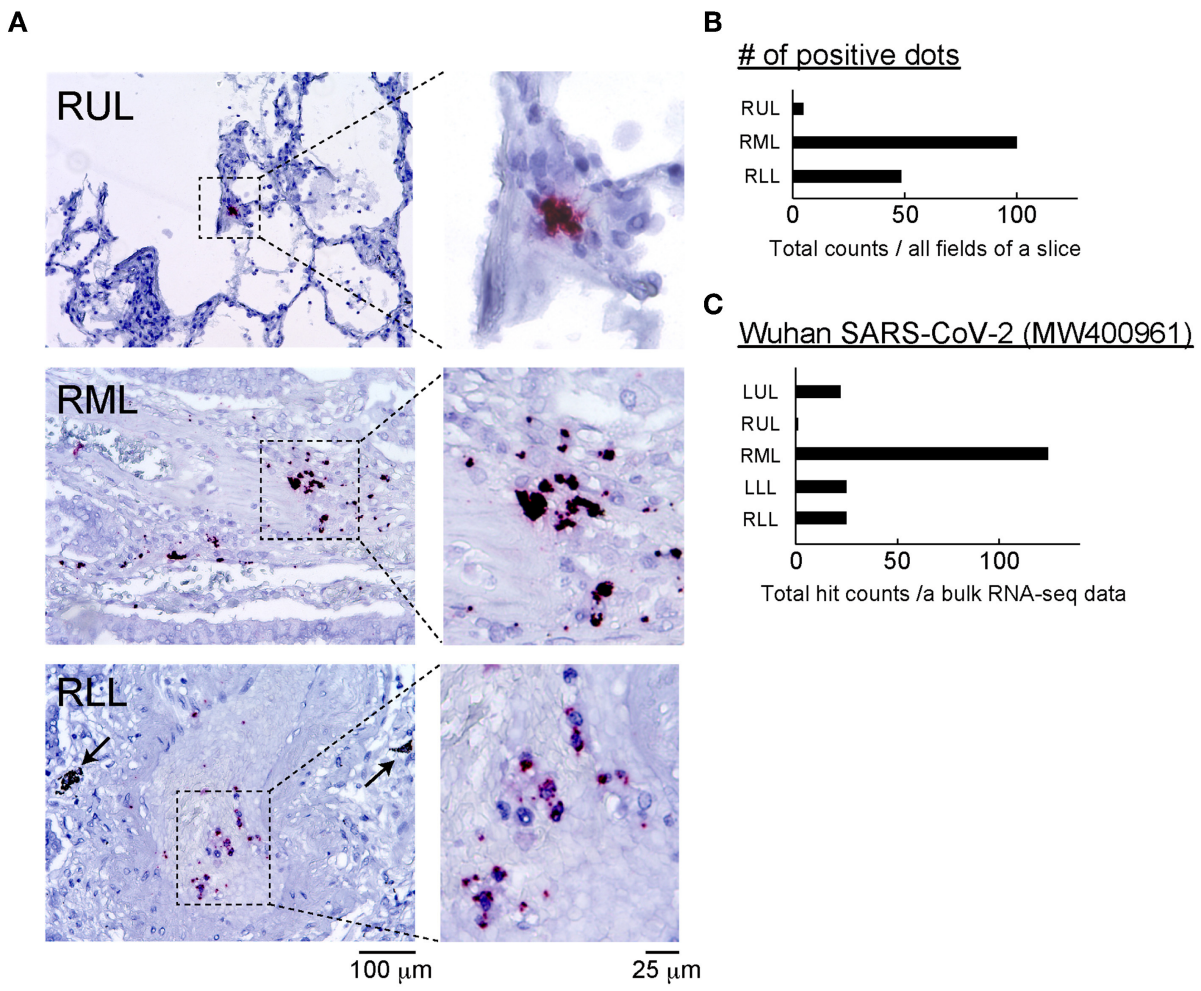
Detection of SARS-CoV-2 and related proteins. **(A)** The ISH assay was performed using two probes: one binding to the SARS-CoV-2 spike protein mRNA (green) and another designed for viral replication (antisense RNA strand, red). Representative positive reactions were shown. There were no green signals, and the red positive dots was limited to blood vessels that infiltrated the alveolar stroma. Scale bars: 100 or 25 μm. **(B)** Positive dots for nCoV2019-S-antisense in all fields of a slide in each lung lobe were counted manually, and the number of positive dots in each site was determined. *n* = 1 of slide in each lung lobe. **(C)** Total count of SARS-CoV-2 gene fragments from each lung lobe annotated to the Wuhan SARS-CoV-2 (MW400961) gene data is shown. The total count of SARS-CoV-2-hitted fragments per sequencing data of bulk RNA-seq was calculated. Bulk RNA-seq data in each lung lobe (*n* = 1) was used.

To identify the SARS-CoV-2 components, the antigens and RNA were analyzed using ISH and bulk RNA-Seq. To visualize SARS-CoV-2 RNA in the FFPE specimens, the ISH assay was performed. The probe for viral replication yielded a positive signal in all lobes (Figure 2A; red dots). All positive signals were counted in the stained slice manually, and the number of positive dots in whole slice was highest in the right middle lobe (RML) tissue (Figure 2B). To confirm the presence of SARS-CoV-2 RNA in this sample, bulk RNA-Seq data from all lung lobes were applied to the Wuhan SARS-CoV-2 complete genome sequencing data. The mapping graph constructed using the sequencing data of RML showed 124 matched fragments with the Wuhan-derived SARS-CoV-2 genomes along with some mismatched or mutated base sequences. The number of matched fragments in the LUL, left lower lobe (LLL), RUL, and right lower lobe (RLL) were 22, 29, 1, and 25, respectively (Figure 2C). These results suggested that the replicating virus could be detected more frequently in the lower lobes than in the upper lobes. Next, we performed IHC analysis for ACE2, TMPRESS2, and FURIN proteins, which support SARS-CoV-2 entry into host cells (Supplementary Figure 1).

### Differences in Immune Cell Counts and IL-6 Production at Different Sites of the Lung Lobes

**Figure 3:**
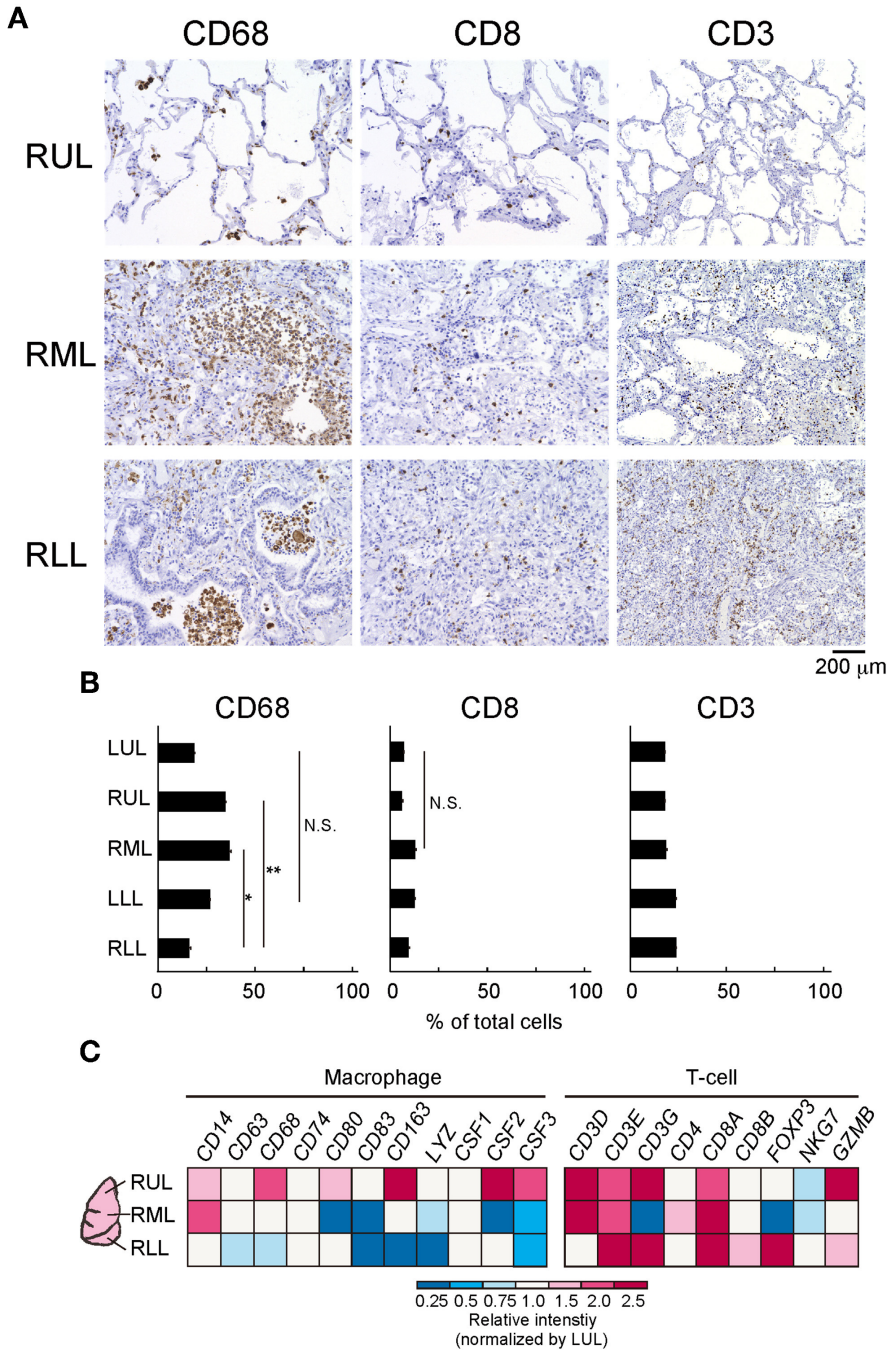
Differences of immune-cells expression at the different site of lung lobes. **(A)** IHC was performed by using each antibody for detecting a macrophage (CD68), T cell (CD8, CD3). Scale bar indicates 200 μm. **(B)** The image analysis by counting positive cells in each site of lung lobe. Six-eight fields per slide from individual images were analyzed. Statistical significance, *t*-test *p < 0.05 or **p < 0.01 compared to the RLL. **(C)** Bulk RNA-Seq analysis of each lung lobe of a patient with COVID-19 with respect to the immune cells present. The colors in the heat map represent the relative intensity (a.u.) normalized by the value obtained in the LUL (1.0, white).

**Figure 4:**
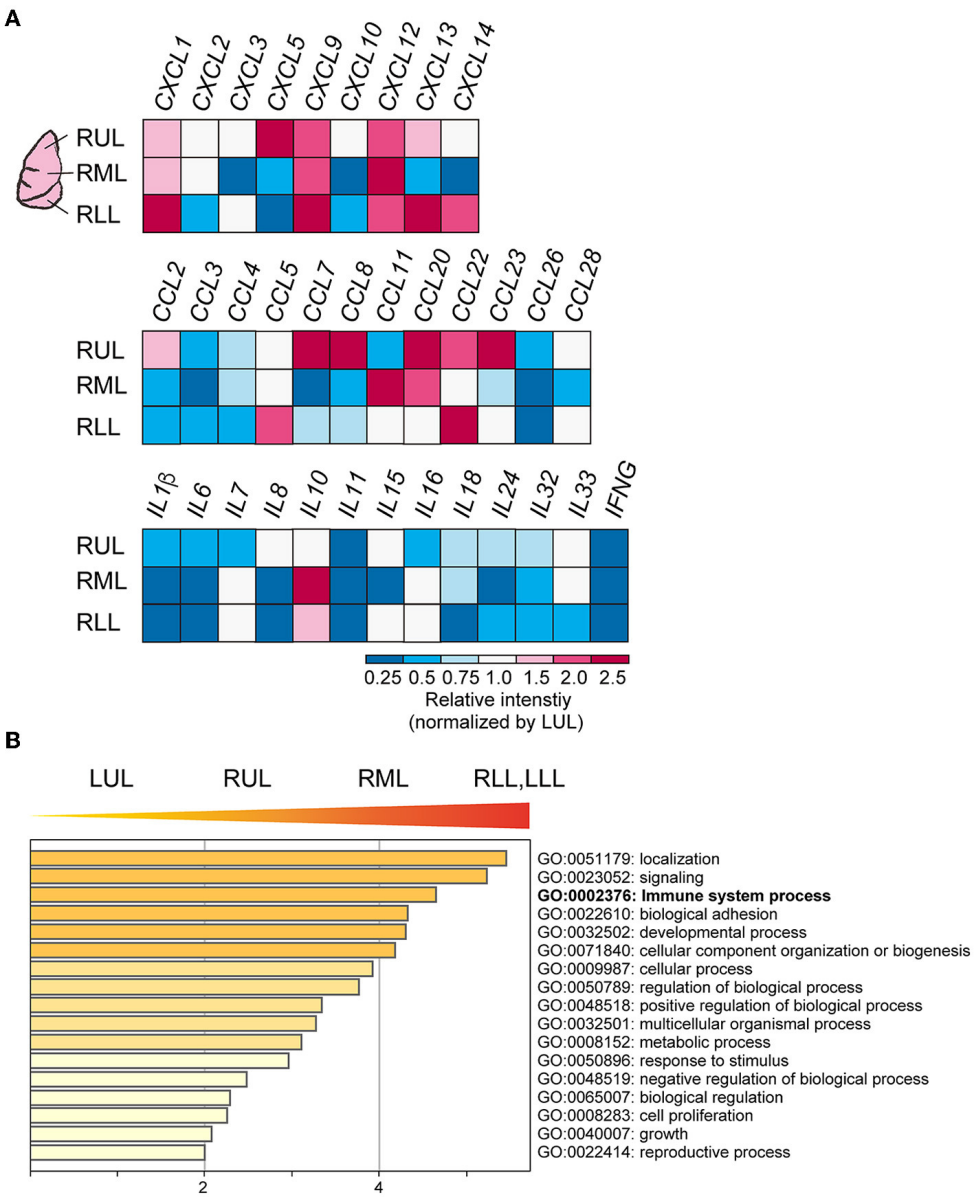
Cytokines and chemokines expression in each site of lung lobes. **(A)** Bulk RNA-Seq data for CXC, CCL families. The relative intensity (a.u.) for each gene was calculated by normalizing the values to those obtained for the LUL. **(B)** Gene enrichment analyses of the DEGs. The higher gene sets that increased as the PG increases. GO terms are labeled with ID and sorted by −log_10_(P) value. The top-level enriched GO biological processes are shown. A darker color indicates as a smaller *p* value (more significant).

To evaluate the immune cell profile in each lung lobe, IHC was performed using antibodies to detect macrophages and T cells (Figure 3A). Image analysis based on averaged counting positive cells per slide showed that the CD68^+^ positive macrophage count increased significantly in the RML (*p* = 0.015) or RUL (*p* = 0.007) as compared to that in the RLL [*F*_(4, 12)_ = 3.259, *p* = 0.0038] (Figure 3B). There were no statistically significant differences in the distribution of CD8^+^ cytotoxic T lymphocytes (*p* = 0.1) and CD3^+^ T cells (*p* =0.15) in the different lung lobes. However, CD8^+^ and CD3^+^ cells tended to increase in lower lung lobes; the average percentages of CD8^+^ and CD3^+^ cells per slice were 6.1 ± 0.03 and 17.7 ± 0.02 (mean ± S.D.) in the RUL and 8.0 ± 0.03 and 23.6 ± 0.02 (mean ± S.D.), respectively in the RLL. As shown in the list of RNAs for identifying macrophages, RNA expression appeared to be enhanced in the RUL (light to dark pink) and suppressed in the RLL (light to dark blue) as compared to that in the relatively normal lung lobe, the LUL (Figure 3C). However, T-cell detection indicated that the expression of each RNA increased considerably in severe inflammatory microenvironments, such as in lower lobes. The mRNA expression of *CXC* and *CCL* families varied, as indicated by the bulk RNA-Seq data for chemokines (Figure 4A). The expression of interleukin families seemed to be decreased in the lower lung lobes. For example, the mRNA expression of *IL-6*, which contributes to excessive inflammation in COVID-19, was decreased in lower lung lobes. The gene expression pattern of macrophages related to mild or severe COVID-19 patients, as previously reported (12), also varied in this patient (Supplementary Figure 2). Moreover, the comprehensive bulk RNA-Seq data were analyzed thoroughly using Metascape (11) to identify the biological pathways that were enhanced at each stage of inflammation. The immune system process (G0: 0002376) was significantly enhanced in lower lung lobes relative to enrichment in upper lung lobes (Figure 4B). A more detailed analysis was performed using Coronascape, a web-based gene annotation system focused on COVID-2019 gene lists (13) (Supplementary Figure 3).

### Basal Cell Outgrowth in Severely Inflamed Lung Lobes

**Figure 5:**
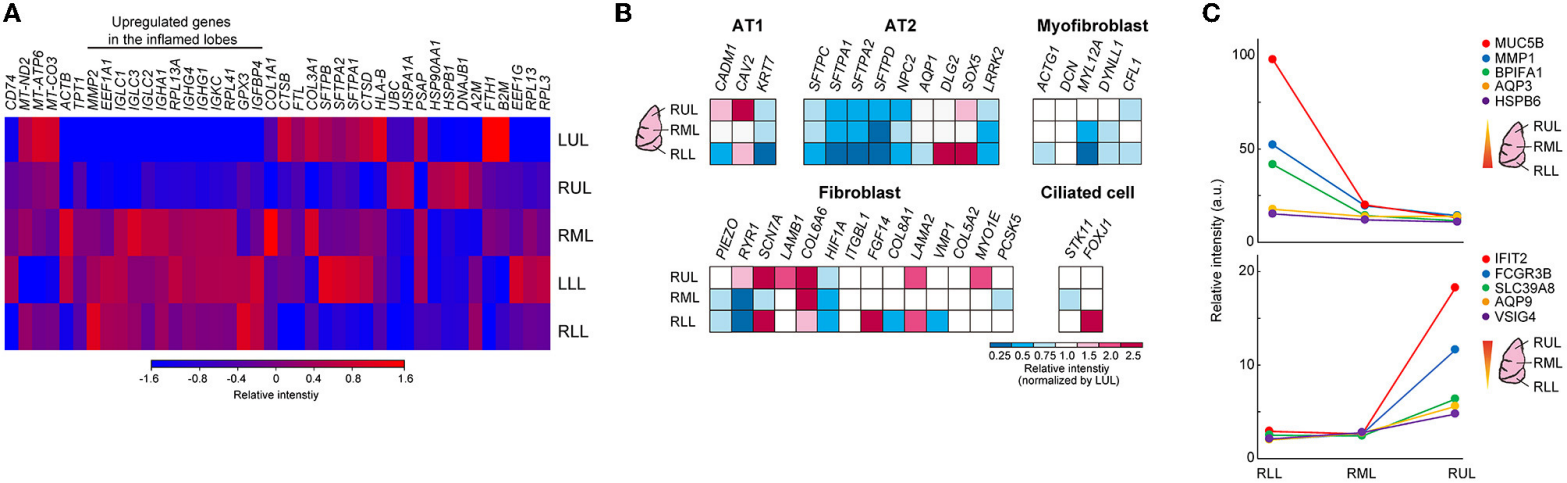
Differences among the different lung lobes microenvironments. **(A)** Heat map clustering at the different site of lung lobes by using bulk RNA-seq data. Each bulk RNA-sq data (*n* = 1) was analyzed. The color bar indicates relative intensity (a.u.). **(B)** Bulk RNA-Seq data for each cell identification marker. Relative intensity (a.u.) normalized to the value obtained in the LUL for each gene (red to blue color bars). **(C)** Time-series analysis by using k-mean clustering test in different inflamed condition was performed, and representative 5 genes were shown in each graph. Top: higher genes in RLL, Lower graph: higher genes in RUL.

A heat map of the objective analysis using bulk RNA-seq data showed the genes that were specifically enhanced, as compared to levels in other sites of lung lobes (Figure 5A). In the severely inflamed lower lobes (RML, LLL, and RLL), mRNA levels for *MMP2, EEF1A1*, and some B-cell receptor domains (*IGLCs, IGHA1, IGHGs, IGKC*) were relatively increased. The prediction of epithelial cell, fibroblast, and ciliated cell populations based on bulk RNA-Seq data showed that the abundance of alveolar type II cells (AT2) was reduced in lower lung lobes, and some type of fibroblasts seemed to be increased in upper lung lobes (Figure 5B). Additionally, gene expression time-series analysis was performed at different sites of the lung lobes, and the five representative genes with the highest expression from RLL to RUL, or the reverse trend, are shown in Figure 5C. The expression of *MUC5B, MMP1, BPIFA1, AQP3*, and *HSPB6* genes tended to be expressed at high levels in severely inflamed lower lung lobes, and *IFIT2, FCGR3B, SLC39A8, AQP9*, and *VSIG4* mRNAs were expressed at higher levels in mildly inflamed upper lung lobes. However, the cell compartments filled with alveolar stromal tissues in lower lung lobes remained unidentified.

**Figure 6:**
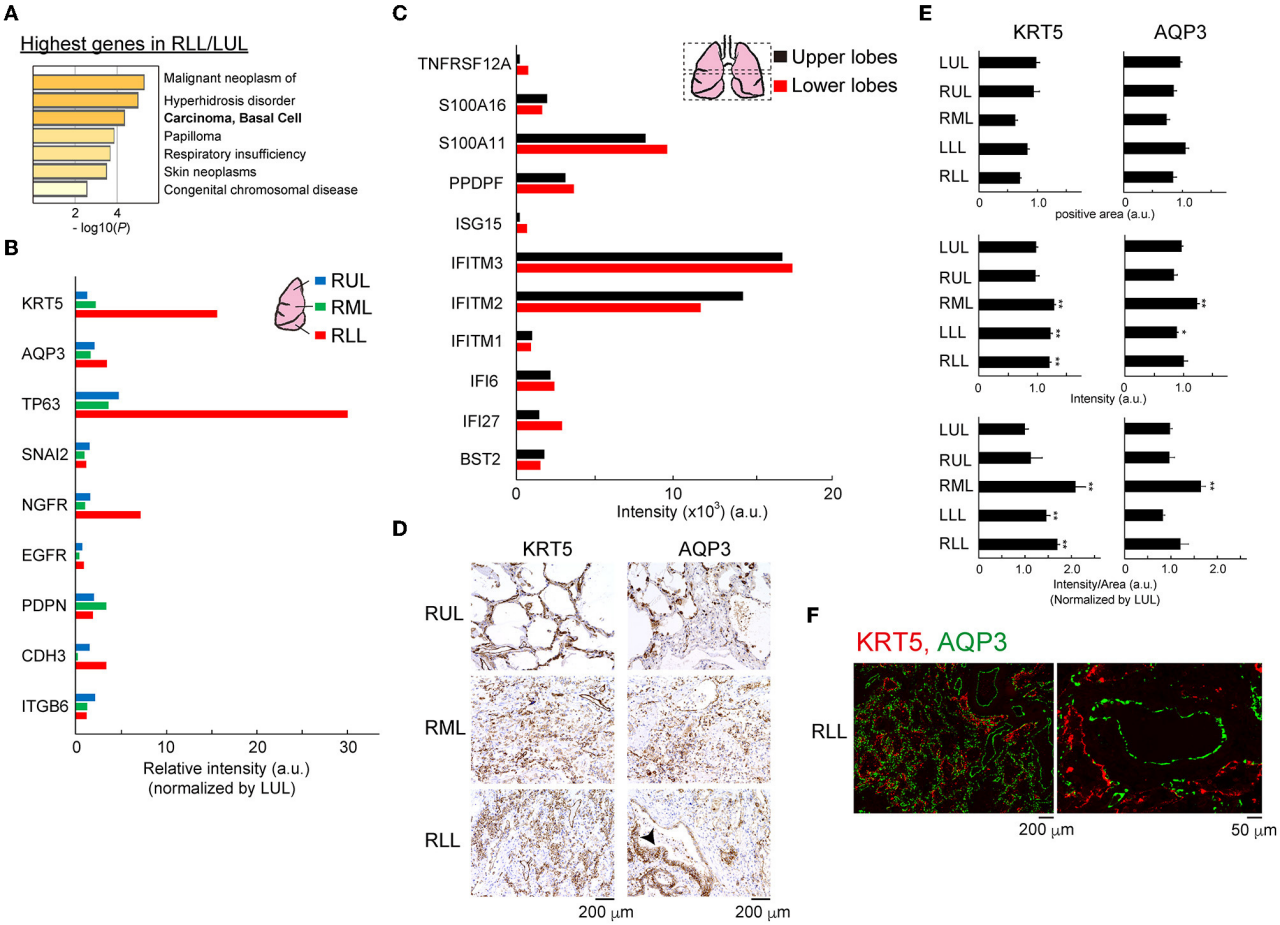
Outgrowth of basal cells in the lower lung lobes. **(A)** Gene enrichment analyses of the DisGeNET. All genes in RLL were normalized by LUL, then the data was applied. **(B)** Bulk RNA-Seq data for basal cell markers in each lung lobe. Relative intensity (a.u.) normalized to the value obtained in the LUL is shown for each gene. **(C)** Bulk RNA-Seq data for intrapulmonary basal-like progenitors (IPBLPs) cell markers. Bulk RNA-seq data from averaged upper lobes (RUL, LUL) and lower lobes (RML, RLL, LLL) were analyzed. **(D)** IHC for KRT5 and AQP3 proteins. Arrowhead indicates restricted staining pattern in the alveoli. Scale bar indicates 200 μm. **(E)** Image analysis for KRT5 and AQP3. Six to eight fields from individual images were analyzed. Mean ± standard deviation. Statistical significance, *t*-test *p < 0.05 or **p < 0.01 vs. LUL. **(F)** Double immunofluorescence staining for KRT5 and AQP3 protein in RLL. Scale bar indicates 200 or 50 μm.

Thus, we focused on the top 10 genes with simply the highest expression normalized by the gene expression value in the lower/upper lung lobe that showed >10-fold changes, and these genes were analyzed using Metascape to predict the development of human diseases in the lower lung lobe microenvironment (Figure 6A) (14). Clinical diagnosis and pathological findings clearly indicated significant similarities to basal cell-related carcinomas. Following this, the gene annotation and analysis resource was used for objective diagnosis depending on the selected genes. Notably, the expression patterns of four of 10 genes (*KRT15, KRT5, CSMD1*, and *FGFR3*) indicated significant similarities to basal cell-related carcinomas. Basal cells are potential stem cells that regulate cell turnover in the airway epithelium (2, 15). Bulk RNA-Seq data related to human basal cell line markers, as shown in Figure 6B, supported the expansion of the basal cell population in lower lung lobes. Especially, the mRNA of the representative basal cell marker keratin 5 (*KRT5*), tumor protein p63 (*TP63*), nerve growth factor receptor (*NGFR*) was strongly expressed in the lower, rather than upper, lung lobe. Eleven representative putative TP63 intrapulmonary basal-like progenitor (IPBLP) cell markers, including *IFI27, IFITM1*, and *S100A11*, have been reported (3, 16); however, our bulk RNA-Seq data showed that the expression level in IPBLPs might be relatively uniform (Figure 6C). An anti-KRT5 antibody was used to confirm whether KRT5 protein was upregulated in the inflamed lung lobes; semi-quantitative image analysis showed that the KRT5-positive response was significantly enhanced in lower lung lobes (Figures 6D,E). Meanwhile, the majority of KRT5-positive staining was observed in the alveolar epithelial cells, and the stained area was spread out. In addition, the aquaporin-3 (AQP3) water channel is a candidate marker of basal-like cells (17); IHC with the anti-AQP3 antibody showed that AQP3-positive staining tended to increase unevenly and was widely distributed in the inflamed lower lung lobes. A restricted staining pattern was observed on the basal membrane of basal-like cells only when the morphology of the alveoli was relatively preserved (Figure 6D, arrowhead). Double fluorescent staining using anti-KRT5 and anti-AQP3 antibodies showed that these proteins were expressed independently (Figure 6F).

## Discussion

Our findings provided an overview of the differences in RNA expression, immune cell populations, cytokine expression, and histopathological characteristics among different sites in the lung lobes of a patient with COVID-19. To the best of our knowledge, the distinct features at different sites of the lung lobes of a patient with COVID-19 have not been analyzed using FFPE samples. In previous studies, SARS-CoV-2 was detected in the hyaline membrane (9), alveolar pneumocytes (5, 18), and alveolar epithelial cells (19), and weak staining was observed in scattered macrophages, but not in ciliated and basal cells (18). Here, the positive signal was limited to blood vessels that infiltrated the alveolar stroma in lower lung lobes, and a similar positive signal was observed in the blood vessels as well as in the stromal organization in upper lung lobes. One macrophage-like cell showed a positive reaction. However, the duration of formalin fixation, antigen inactivation methods, duration of treatment, and antibody dilution influence the quality of ISH using FFPE samples; therefore, the identification of SARS-CoV-2-positive cells requires careful interpretation. SARS-CoV-2 was detected most frequently in the moderately inflamed middle lung lobe rather than in the severe inflamed lower lung lobes. The initial stage of SARS-CoV-2 infection progressed from upper to lower lobes, and it was likely delivered to a greater extent to the lower lung lobes which had thin bronchi. Subsequently, SARS-CoV-2 was eliminated by an immune response, but an excessive inflammatory response was occurred in lower lobes. The remaining SARS-CoV-2 virus and moderate inflammatory response observed in the middle lung lobe may be an intermediate stage. Since the events initiated by SARS-CoV-2 in the lung lobes at a certain time point were observed in FFPE tissues, the virus localization and inflammatory responses at each site provided various important indications.

SARS-CoV-2 enters host cells, such as respiratory epithelial cells, by binding to ACE2, which is a type I transmembrane glycoprotein with carboxypeptidase activity; however, the abundance and distribution of ACE2 in human lungs are elusive. For instance, ACE2 is expressed at high levels in alveolar type II cells (AT2s) (20), which are rare ciliated epithelial and endothelial cells present in the trachea, but not in the lungs (21). In other studies, AT2 as well as ciliated cells in human lungs were shown to express ACE2 (22). There is no significant difference in ACE2 expression between healthy individuals and patients with chronic respiratory disease (23), but ACE2 is expressed at high levels in the lungs of patients with specific lifestyle habits, such as a smoking habit (24). Here, ACE2 was expressed at high levels in lower than in upper lung lobes. Cellular population analysis based on bulk RNA-Seq data indicated that the AT2 cells tended to decrease in lower lung lobes, speculating that not AT2 but other cells highly expressed ACE2 proteins after removal of SARS-CoV-2 or the lung tissue had collapsed owing to SARS-CoV-2 infection. Recent genome-wide screening against SARS-CoV-2 indicates that the enhancement of glycosaminoglycan and glycosylphosphatidylinositol biosynthesis, sterol regulatory element-binding protein and bone morphogenetic protein signaling in host cells are required for infection (25); but there were no clear differences in the distribution of identified genes throughout lung lobes in this patient (Supplementary Figure 4).

Single-cell RNA-Seq analysis of bronchial tissue from patients with COVID-19 also indicated that the number of resident macrophages, regulatory T cells, cytotoxic T cells, and B cells decreased significantly in patients (13). Here, the analysis did not involve comparison to healthy samples, and it was unknown whether the total CD8^+^ T cell count in the peripheral blood and lung tissues decreased. Our results demonstrated that the number of CD68^+^ macrophages and CD14^+^ monocytes increased in upper-to-middle lung lobes with mild-to-moderate inflammation, and the number of CD8^+^ and CD3^+^ T cells tended to increase in middle-to-lower lobes with moderate-to-severe inflammation. This indicates that viral infection and elimination by a series of immune responses could be observed in a single lung tissue. In general, the number of CD4^+^ and CD8^+^ T cells in lymph nodes decrease in severe COVID-19; therefore, these cells may also decrease in the peripheral blood (26). Our analysis did not involve comparison to healthy samples, and it was unknown whether the total CD8^+^ T cell count in the peripheral blood and lung tissues decreased. Importantly, the CD14^+^ monocytes in patients with COVID-19 demonstrate that inflammatory monocytes induce pathogenic T cells, thereby promoting an inflammatory cytokine storm (27). In addition, the number of neutrophils increases in severe COVID-19 and has been associated with poor prognosis (13, 26, 28, 29). Pathological analysis using H&E and Masson's trichrome staining showed that the neutrophil count increased in the middle-to-lower lung lobes.

The differences between the plasma cytokine and chemokine levels in healthy donors and patients with COVID-19 have been reported in studies (26, 30–32). The mRNA expression of most interleukins and IFNs was highest in the LUL, indicating that the upper lung lobes were the forefront of the biological defensive responses against SARS-CoV-2. Serum IL-6 elevation is observed in severe cases of COVID-19, and excessive IL-6 released from macrophages triggers a cytokine storm and leads to immune exhaustion (31–36). Therefore, the blockade of IL-6 or its receptor using drugs such as tocilizumab can be a potential therapeutic strategy for COVID-19. However, the results of clinical trials are controversial, and no conclusions have been made thus far (37–39). Our results indicated that the highest IL-6 mRNA levels were not in the severe but mild inflamed tissue, implying that early treatment with glucocorticoids and IL-6 antagonists for mild inflammation could be beneficial against COVID-19; however, the treatment is not useful once activated macrophages cause the IL-6-induced cytokine storm. This is a valuable finding from the comprehensive analysis of the lung lobe microenvironment of the patient.

The primary indication in the lung histopathology of patients with severe COVID-19 was diffuse DAD with hyaline membrane formation resulting in the desquamation of pulmonary epithelial cells and serous exudation into the alveolar space (40–42). Interstitial mononuclear inflammatory cells, predominantly lymphocytes, which easily infiltrated the space, were observed during the pathological analysis of the lower lung lobes. Overall, an autoimmune reaction caused by SARS-CoV-2 infection induced an inflammatory reaction first in the lower lung lobes, followed by excessive angiogenesis, immune cell infiltration and a cytokine storm induced by some cytokines such as IL-6. Usually, as infection progresses, fibrosis is induced and further promoted by fibroblast proliferation, resulting in an increase in granulation and filling of the alveolar space and stroma tissue. Masson's trichrome staining and the bulk RNA-Seq data indicated that fibroblast proliferation and fibrosis were promoted, although not remarkably. Metascape analysis predicted the chances of malignant neoplasm of the salivary gland, hyperhidrosis disorder, carcinoma of basal cells, papilloma, respiratory insufficiency, skin neoplasms, and congenital chromosomal disease in lower lung lobes. This prompted us to investigate the possibility of abnormal basal cell biological processes in the lower lung lobes.

The lung can regenerate by proliferation and differentiation of resident progenitor cell, basal cells upon injury. AT2 cells can self-renew and give rise to AT1cell which can gas exchange in the lung, and the disruption of AT1 and AT2 cells induces DAD (43, 44). It has been suggested that KRT5^+^TP53^+^ basal cells may differentiate into AT2 cells. Thus, basal cells are candidate cells that cover exposed alveoli and seal any leakage; these multipotent adult tissue stem cells are involved in the pathology of SARS-CoV-2 infection (2, 15). Single-cell RNA-Seq data from 19 clinically well-characterized patients with COVID-19 showed that the ratio of basal cells to total epithelial cells was higher in patients with moderate rather than critical COVID-19 (13). Based on our findings, one of the potential reasons for the lack of respiratory failure improvements in the patient was the excessive self-renewal of basal cells, which led to basal cell hyperplasia. Previous reports showed that basal cells might not be infected with SARS-CoV-2 (18). Airway basal cells differentiate into multipotent progenitor or luminal progenitor cells, which leads to *KRT5, TP63*, and *NGFR* downregulation (15); however, these three genes were upregulated in lower lung lobes, and their expression was proportional to the structural deterioration. This means that undifferentiated airway basal cells were increased in lower lung lobes. Public single-cell RNA-Seq data of nine patients with COVID-19 revealed that in severe cases, cell clusters of lung progenitors contain a high number of KRT5-positive cells, which help establish the epithelial barrier to prevent leucocyte-mediated cytotoxicity (2). Putative intrapulmonary basal-like progenitors (IPBLPs), which differ from KRT5^+^/TP63^+^ cells, were shown to contribute to regeneration of the alveolar epithelial barrier (3, 16). It was speculated that the number of AT2 cells would reduce and the KRT8^+^ pre-alveolar type (PATS) 1 transitional cell state (PATS/ADI/DATP), which may represent IPBLP-like cells, would be upregulated (16). In COVID-19 alveoli, AT2 cell self-renewal are inhibited and PATS and IPBLPs accumulation are observed, implying the impairment of normal functional improvement in alveolar epithelial cells. Here, the expression of AT2 cell and IPBLP markers was not changed throughout lung lobes, indicating that renewal of the alveolar epithelial barrier had occurred in all lung lobes, and some normal basal cell regeneration might occur. The functions of basal cells expressing different genes in severe pathological lung environments remain unknown. We hypothesized that in this patient, the basal stem cells (KRT5^+^ and/or TP63^+^) or AQP3^+^ basal-like cells might have lost their differentiation ability and only filled the collapsed alveolar space but could not regenerate the alveolar epithelial tissue, which led to the loss of alveolar structure and function.

AQP3 facilitates the membrane uptake of glycerol and hydrogen peroxide (45), and it is specifically expressed in the basal airway epithelial cells of the human trachea (17). It is widely accepted that AQP3 contributes to the high glycerol and ATP supply for cancer cells required for cell growth maintenance (46–49). For example, AQP3 overexpression in AQP3-PC12 cells and epidermoid carcinoma cells (A431), which express high levels of AQP3, accelerates cell cycles, and treatment with the AQP3 inhibitor leads to arrest in the S-G2/M phases and enhances the expression of cyclin D1 and E1 proteins (47, 48). Moreover, AQP3 promotes the expression of CD44, a cancer stem marker, through the Wnt/β-catenin signaling pathway in gastric cancer cells (49). The damaged lung lobes expressed *AQP3* mRNA and proteins at high levels, and AQP3^+^ cells were completely distinguishable from KRT5^+^ cells since we did not detect double-positive cells. In addition, the expression patten of AQP3 protein and SARS-CoV-2 in each site of the lung lobes were similar. AQP3 neutralization helps to suppress liver injury and fibrosis regulated by macrophages and might be a useful therapeutic strategy for oxidative stress-related diseases (50). Thus, our hypothesis is that not only KRT5^+^TP63^+^ basal cells, but also AQP3^+^ basal-like cells, proliferated abnormally to fill the alveolar space and stroma tissue that was collapsed upon SARA-CoV-2 infection, and suppression of the increase in KRT5^+^TP63^+^ basal cells and/or AQP3^+^ basal-like cells might retain a normal inflammatory defending response and facilitate the regeneration of airway alveolar epithelial cells. Further experiments on SARS-CoV-2 infection in human lung organoids, with KRT5-positive basal cells constituting ~10% of the cells (44), are warranted to confirm the effect of basal cells and/or basal-like cell inhibition.

In conclusion, our comprehensive analysis of the lung lobes of a patient with COVID-19 indicated SARS-CoV-2 enrichment and the cellular and molecular differences among lung microenvironments with mild-to-severe inflammation. We speculated if this patient survived more, pneumonia symptoms would have continued to progress in all lung lobes. Thus, our study describing the detailed analysis of different sites of the pathological tissue from one patient might contribute to our understanding of viral transmission and inflammation from the pre-illness to disease-onset stages in other pandemics in the future.

## Data Availability Statement

The datasets presented in this study can be found in online repositories. The names of the repository/repositories and accession number(s) can be found in the article/Supplementary Material.

## Ethics Statement

This study was approved by the Research Ethics Committee of Wakayama Medical University (approval no. 2882). Written informed consent for participation was not required for this study in accordance with the national legislation and the institutional requirements.

## Author Contributions

SI, KM, MH, MK, TK, HY, and SH conceived the experiment. KM and TK obtained samples and prepared the FFPE blocks. YM and SM performed IHC data analysis. SI, MH, and SH performed other experiments and analyses. KM, MK, SM, TK, and HY interpreted the data. SI and SH wrote the paper with contributions from all authors. All authors contributed to the article and approved the submitted version.

## Funding

This work was supported by JST CREST Grant Number JPMJCR5G3.

## Conflict of Interest

The authors declare that the research was conducted in the absence of any commercial or financial relationships that could be construed as a potential conflict of interest.

## Publisher's Note

All claims expressed in this article are solely those of the authors and do not necessarily represent those of their affiliated organizations, or those of the publisher, the editors and the reviewers. Any product that may be evaluated in this article, or claim that may be made by its manufacturer, is not guaranteed or endorsed by the publisher.
