# Supplementary material for: Immune Cells Profiles in the Different Sites of COVID-19-Affected Lung Lobes in a Single Patient
Source: Front Med (Lausanne). 2022 Feb 16;9:841170. doi: 10.3389/fmed.2022.841170 (PMC8888686; doi:10.3389/fmed.2022.841170)
Supplement: Supplementary file 1 [file Data_Sheet_1.docx]

**Immune cells profiles in the different sites of COVID-19-affected lung lobes**

Sadahiro Iwabuchi^1^, Kyohei Miyamoto^2^, Mayuko Hatai^1^, Yurina Mikasa^3^, Masahiro Katsuda^4^, Shin-ichi Murata^3^, Toshikazu Kondo^5^, Hiroki Yamaue^4^, and Shinichi Hashimoto^1^*

^1^Department of Molecular Pathophysiology, Institute of Advanced Medicine, Wakayama Medical University, 811-1, Kimiidera, Wakayama city, Wakayama, 641-8509, Japan

^2^Department of Emergency and Critical Care Medicine, Wakayama Medical University, 811-1, Kimiidera, Wakayama city, Wakayama, 641-8509, Japan

^3^Departments of Human Pathology, Wakayama Medical University, 811-1, Kimiidera, Wakayama city, Wakayama, 641-8509, Japan

^4^Second Department of Surgery, Wakayama Medical University, 811-1, Kimiidera, Wakayama city, Wakayama, 641-8509, Japan

^5^Department of Forensic Medicine, Wakayama Medical University, 811-1, Kimiidera, Wakayama city, Wakayama, 641-8509, Japan

*Corresponding author:

Shinichi Hashimoto, Ph.D.

Department of Molecular Pathophysiology, Institute of Advanced Medicine, Wakayama Medical University, 811-1 Kimiidera, Wakayama city, Wakayama, 641-8509, Japan

Telephone: +81-73-441-0907

Fax: +81-73-441-0908

Email address: [hashimot@wakayama-med.ac.jp](mailto:hashimot@wakayama-med.ac.jp)

**Supplemental Table.1**

**Pathological evaluation**

| Grade | Definition | Lung Lobes |
| --- | --- | --- |
| 4 | Completely organized lung. Clear alveolar cavity is not observed. | RLL, LLL |
| 3 | Severely inflamed and organized lung. Alveolar stromal tissue is almost organized, but a few parts of the alveolar cavity are open (<30%) | RML |
| 2 | Moderately inflamed and organized lung. Alveolar stromal tissue has fibrosis and organization, and alveolar cavity is partially open (30%– 70%) | RUL |
| 1 | Mildly inflamed and organized lung. Alveolar stromal tissue has fibrosis, but alveolar cavity is almost open (>70%), | LUL |
| 0 | Normal lung. Alveolar cavity is completely open | - |

Abbreviations: RLL, right lower lobe; RML, right middle lobe; RUL, right upper lobe, LUL, left upper lobe; LLL, left lower lobe

**Supplemental Figures and Figure legends**

**
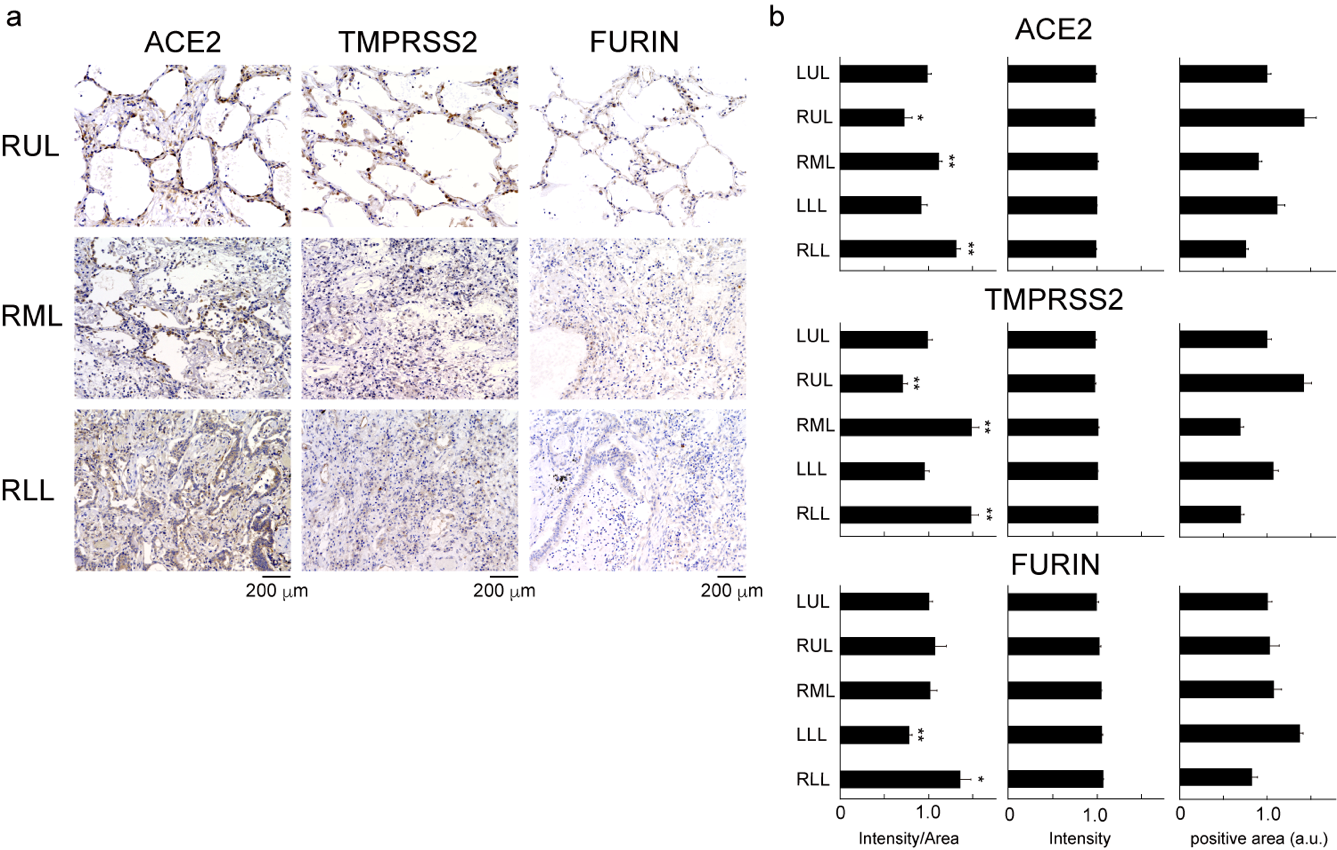
**

**Supplemental Fig 1. Immunohistochemistry of ACE2, TMPRSS2 and FURIN proteins.**

(a) IHC by using ACE2, TMPRSS2 and FURIN antibodies. Scale bar indicates 200 μm. (b) Semiquantitative image analysis of these proteins. The relative intensity value (intensity/area) of ACE2 and TMPRSS2 was significantly higher in the RML and RLL as compared to that in the LUL. FURIN expression was significantly higher in the RLL but lower in the LLL. **p* < 0.05 or ***p* < 0.01 vs each LUL.


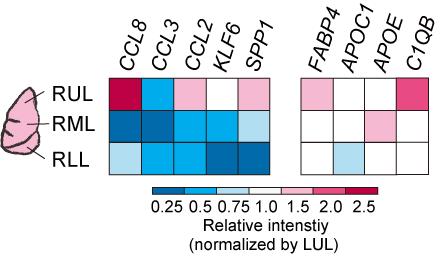


**Supplemental Fig 2. Bulk RNA-seq data related to macrophages in previous report.**

Shaath et al. Cells 2020 describe that macrophage in severe COVID-19 highly expressed *CCL8*, *CCL3*, *CCL2*, *KLF6*, *SPP1* and the macrophage in mild COVID-19 patient has different gene signatures (*FABP4*, *APOC1*, *APOE*, *C1QB*). Our bulk RNA-seq data could not detect the similar gene expression pattern in different site of lung lobes from a patient.


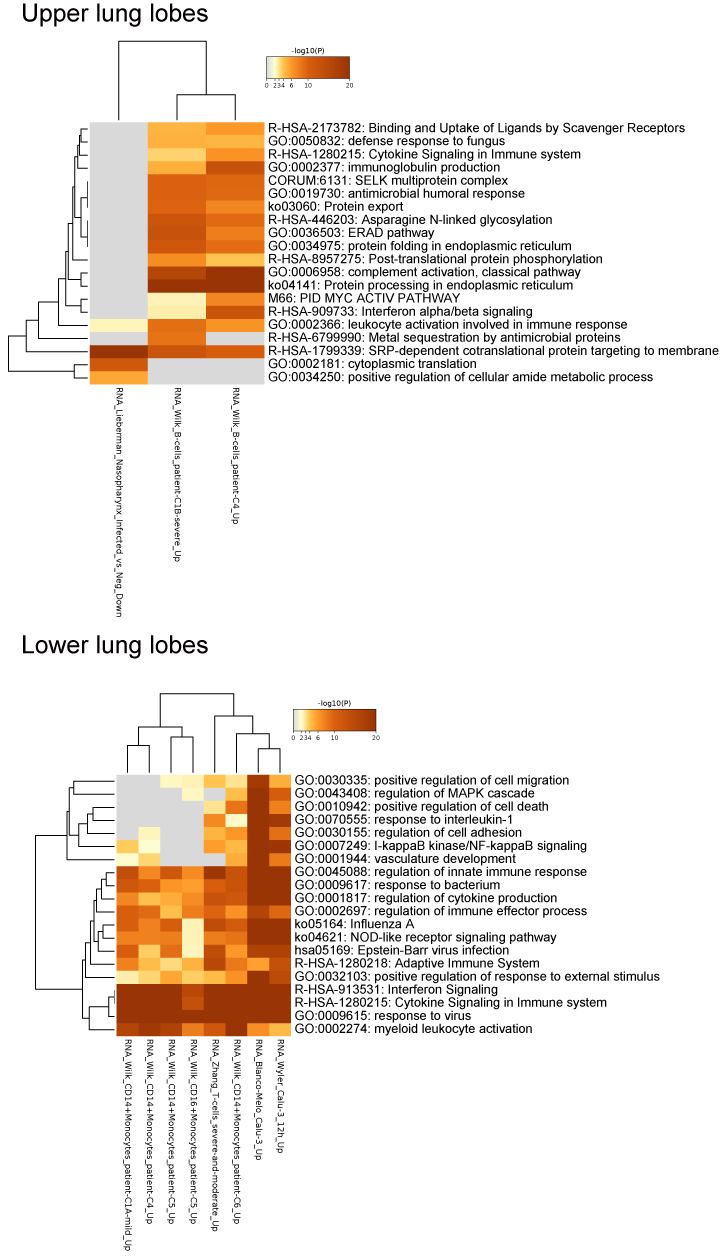


**Supplemental Fig 3. Gene enrichment analyses by using Coronascape.** The higher gene sets in RLL and LUL sample applied to selected COVID-19 reference lists. GO terms are labeled with ID. The scale bars indicate -log_10_(P) value, and a darker color indicates as a smaller *P* value. The top 20 enriched GO terms are shown. At least eight data sets related to COVID-19 were significantly similar (logP = -9.02 to -16.89) to the data in lower lung lobes.


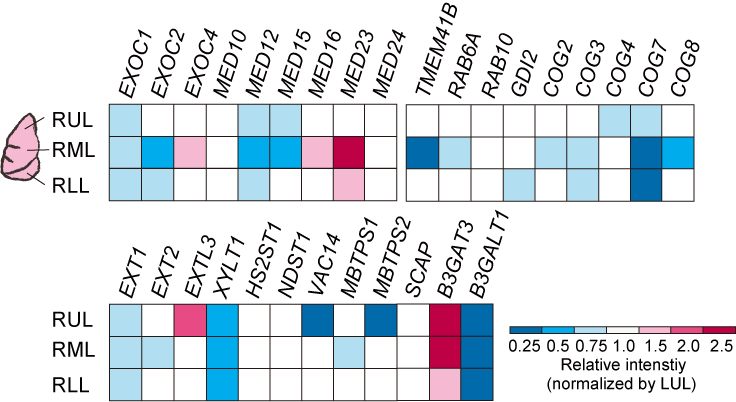


**Supplemental Fig 4. Bulk RNA-seq data related to SARS-CoV-2 host cells in previous report.**

Schneider et al. Cell 2021 identify the host factors and pathways co-opted by SARS-CoV-2, and the gene sets are related to virus-specific functional roles, including major dependency on glycosaminoglycan or glycosylphosphatidylinositol biosynthesis, sterol regulatory element-binding protein or bone morphogenetic protein signaling. In addition, they find TMEM64 domain-containing protein transmembrane protein 41B (*TMEM41B*) is the top-scoring host factor critical for infection by SARS-CoV-2. However, our bulk RNA-seq data did not provide a unified view.
